# Characterizing low affinity epibatidine binding to α4β2 nicotinic acetylcholine receptors with ligand depletion and nonspecific binding

**DOI:** 10.1186/2046-1682-4-19

**Published:** 2011-11-23

**Authors:** Alexandra M Person, Gregg B Wells

**Affiliations:** 1 Department of Molecular and Cellular Medicine, Texas A&M University System Health Science Center, College Station, TX 77843-1114, USA; 2 Department of Neuroscience and Experimental Therapeutics, Texas A&M University System Health Science Center, Bryan, TX 77807-3260, USA; 3 Department of Veterinary Pathobiology, Texas A&M University, College Station, TX 77843-4467, USA

## Abstract

**Background:**

Along with high affinity binding of epibatidine (*K*_d1_≈10 pM) to α4β2 nicotinic acetylcholine receptor (nAChR), low affinity binding of epibatidine (*K*_d2_≈1-10 nM) to an independent binding site has been reported. Studying this low affinity binding is important because it might contribute understanding about the structure and synthesis of α4β2 nAChR. The binding behavior of epibatidine and α4β2 AChR raises a question about interpreting binding data from two independent sites with ligand depletion and nonspecific binding, both of which can affect equilibrium binding of [^3^H]epibatidine and α4β2 nAChR. If modeled incorrectly, ligand depletion and nonspecific binding lead to inaccurate estimates of binding constants. Fitting total equilibrium binding as a function of total ligand accurately characterizes a single site with ligand depletion and nonspecific binding. The goal of this study was to determine whether this approach is sufficient with two independent high and low affinity sites.

**Results:**

Computer simulations of binding revealed complexities beyond fitting total binding for characterizing the second, low affinity site of α4β2 nAChR. First, distinguishing low-affinity specific binding from nonspecific binding was a potential problem with saturation data. Varying the maximum concentration of [^3^H]epibatidine, simultaneously fitting independently measured nonspecific binding, and varying α4β2 nAChR concentration were effective remedies. Second, ligand depletion helped identify the low affinity site when nonspecific binding was significant in saturation or competition data, contrary to a common belief that ligand depletion always is detrimental. Third, measuring nonspecific binding without α4β2 nAChR distinguished better between nonspecific binding and low-affinity specific binding under some circumstances of competitive binding than did presuming nonspecific binding to be residual [^3^H]epibatidine binding after adding a large concentration of cold competitor. Fourth, nonspecific binding of a heterologous competitor changed estimates of high and low inhibition constants but did not change the ratio of those estimates.

**Conclusions:**

Investigating the low affinity site of α4β2 nAChR with equilibrium binding when ligand depletion and nonspecific binding are present likely needs special attention to experimental design and data interpretation beyond fitting total binding data. Manipulation of maximum ligand and receptor concentrations and intentionally increasing ligand depletion are potentially helpful approaches.

## Background

Ligand depletion can significantly affect estimates for dissociation (*K*_d_) or inhibition (*K*_i_) constants from equilibrium binding data of epibatidine (EB) and α4β2 nicotinic acetylcholine receptor (nAChR) because of the high affinity of EB (*K*_d1_≈10 pM). Errors from ligand depletion arise from inappropriately assuming that free ligand concentration equals total ligand concentration while using total ligand concentration as the independent variable for modeling the binding data. The assumption is attractive because total ligand concentration as the independent variable is suitable for least squares fitting of binding data [1,2]. Ligand depletion can be minimized when designing binding experiments with EB and α4β2 nAChR. Radiolabeled EB with higher specific activity (for example, ^125^I instead of ^3^H) can lead to less ligand depletion by allowing a smaller concentration of α4β2 nAChR to produce useful data. A larger reaction volume at a fixed mole quantity of α4β2 nAChR reduces ligand depletion by reducing the difference between free and total concentration of radiolabeled EB. These avoidance strategies based on design of experiments, however, might be difficult to use in some situations. For example, a newly developed and ^3^H-labeled EB derivative might be available only with low specific activity. Large reaction volumes might be impractical for numerous samples associated with high throughput screening [3]. When ligand depletion cannot easily be avoided, how can data with both ligand depletion and nonspecific binding (NSB) be correctly interpreted from EB and α4β2 nAChR?

Effects of ligand depletion on binding data have long been recognized, leading to models that correctly include ligand depletion with single and multiple specific binding sites [3-9]. For [^3^H]EB, a ligand with relatively low specific activity, and α4β2 nAChR, ligand depletion has been recognized and avoided as a potentially confounding factor for interpreting binding data [10-17]. Alternatively, one site and two sites models for estimating binding constants have included ligand depletion with negligible NSB [18]. Combining ligand depletion and NSB, however, imposes additional demands on binding models. For example, specific binding cannot be calculated simply by subtracting NSB from total binding. Instead, a binding model including both ligand depletion and NSB must fit total binding [6] as has been shown with one specific binding site [19]. In addition to the high potency or high affinity site, functional data from electrophysiology and ^86^Rb^+ ^flux [20-41] and binding data [12,18,20,42,43] for α4β2 or α4β2-containing nAChR suggest a second, low potency or low affinity specific binding site. The difference in agonist potency at the two sites in functional assays has been attributed to α4β2 nAChR with different α:β stoichiometries [21,25,28,30,33]. (α4)_2_(β2)_3 _contributes high potency and (α4)_3_(β2)_2 _contributes low potency. Binding data from our laboratory suggest two independent sites and not two cooperative sites [18]. The physical basis of low affinity equilibrium binding of [^3^H]EB detected under some conditions and the relationship between the low affinity site observed by equilibrium binding and the low potency site observed by functional methods are not known. On the other hand, a single binding site has been suggested for α4β2 nAChR [12,14] and for extracellular domain α4β2 nAChR [18] from binding data. Photolabeling of α4β2 with [^3^H]EB also identified a single binding site [44]. Models of α4β2 nAChR binding data, therefore, should not assume the presence of high and low affinity sites. Instead, an interpretation of binding data needs to test the hypothesis of one binding site versus more than one binding site.

How does interpreting binding data with ligand depletion with NSB and a single binding site [19,45] need to be modified when a second, low-affinity specific binding site might be present from α4β2 nAChR? Detecting and accurately interpreting properties of the low affinity site is important because of the potential biological relevance of the low-affinity specific site. The low affinity binding site might reflect biologically important roles for α4β2 nAChR, reflect a variant structure of the agonist binding site, or give insight into the assembly of α4β2 nAChR. The goal of this study was to determine, using computational modeling, whether fitting total binding is sufficient for characterizing the low affinity binding site from α4β2 nAChR in the context of ligand depletion and NSB. The modeling simulated saturation binding, homologous competition, and heterologous competition. The experimental foundation for the modeling was reported previously with *K*_d1 _= 13 pM for the high affinity site and *K*_d2 _= 12 nM for the low affinity site [18]. The findings are potentially relevant to other ligands and receptors when two or more specific binding sites are possible and when ligand depletion and NSB affect binding data.

## Methods

### Equations of the models

**Figure 1:**
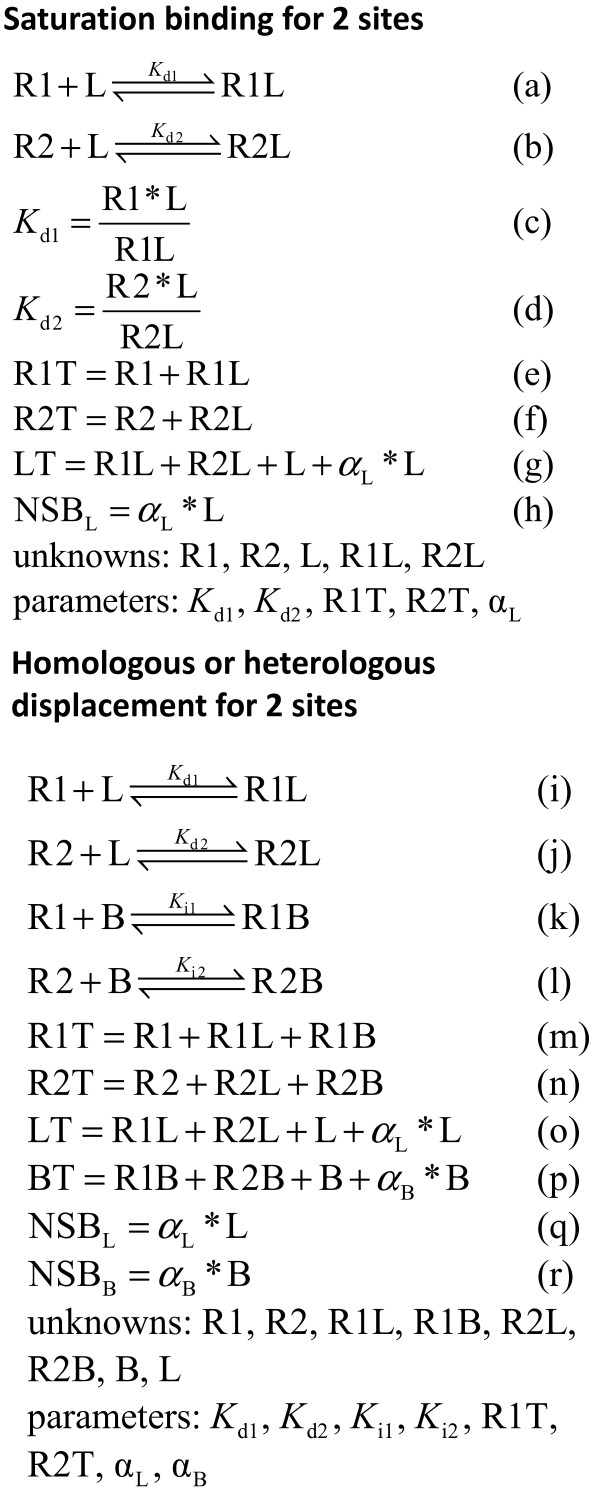
**Equations for the binding models are based on the law of mass action and conservation of mass**. Two mass action equations (c)-(d) for dissociation constants derived from (a)-(b) and three conservation of mass equations (e)-(g) formed the five equations solved simultaneously for the two sites model_total _for saturation binding. Four mass action equations for dissociation constants derived from (i)-(l) and four conservation of mass equations (m)-(p) formed the eight equations solved simultaneously for the two sites model_total _for homologous or heterologous competition. L was the independent variable for two sites model_free _for saturation binding, which did not include Eq. (g). B was the independent variable of two sites model_free _for competition, which did not include Eq. (p). One site model_total _excluded terms referring to the second site. The two sites model for saturation binding that ignored ligand depletion was based on Eqs. (c)-(f) and assumed LT = L. Notation: α_L _= constant describing NSB of radioligand; *K*_d1 _= dissociation constant of high affinity binding site; L = free radioligand; LT = total radioligand; NSB_L _= nonspecific binding of radioligand; R1 = unbound first binding site; R1L = radioligand bound to first site; R1T = total high affinity binding site; B = free competitor (blocker). With analogous notations, the index "2" in these equations refers to the low affinity binding site.

For an introduction to interpreting equilibrium binding with ligand depletion and NSB, see Swillens [19] and Motulsky and Christopoulos [7]. The models of saturation binding and homologous and heterologous competition were based on mass action equations and conservation of mass (Figure 1). Figure 1 shows the notations for the states and equations for the equilibrium dissociation and inhibition constants of the models. Equations for a model were solved numerically within a Microsoft Excel environment using the Maple version 13 or 14 (Maplesoft) add-in. Parameters of a model were optimized to simulated data with the method of least squares using Excel and the Premium Solver Platform (Frontline Systems). Values of parameters were constrained to physically valid values.

Analytical solutions of cubic equations are available that describe ligand depletion (with and without NSB) of two binding sites and one ligand or of two sites with homologous competition [3,9,18,46,47]. Analytical solutions of a quartic polynomial describing ligand depletion and NSB of three binding sites and one ligand or of two binding sites with homologous competition can be derived from the general solution of a quartic polynomial [48]. Numerical solutions were used in this investigation because of the relative ease of implementation and the usefulness of numerical solutions when roots of quintic and higher order polynomials are needed to describe ligand depletion but for which analytical solutions are not available. For example, roots of a sixth order polynomial are needed to describe heterologous competition with two sites and ligand depletion and NSB, which precludes an analytical solution.

### Data generation and model fitting

True binding behaviors (i.e., noiseless data) were defined as the output from two-sites models using defined values of parameters and free ligand concentration as the independent variable (two sites model_free_). Noise was superimposed by adding, to each noise-free data point, a random number selected from a standard normal distribution with a constant standard deviation (SD) determined by context. The SD value was constant along the *x*-axis. In some cases, noise was described by the maximum signal to noise ratio (S/N). The SD for noise was the maximum signal in the noiseless data divided by the maximum signal to noise ratio (i.e., SD = (maximum signal)/(stated maximum S/N)). Multiple data sets with different SD values for noise were fitted simultaneously by weighting, by the inverse of the variance of the noise, the contribution of a data set to the sum of squares. Total concentration of added ligand was the independent variable for the one site model_total _and two sites model_total _when fitting noiseless and noisy data that included NSB. All results are displayed using total concentration on the *x*-axis. All ligand concentrations appearing in the text refer to total concentration unless otherwise noted. The two sites model for saturation binding that ignored ligand depletion assumed that LT = L. The two sites model of apparent specific saturation binding was based on equations (c)-(g) and assumed α = 0 in equation (g) (Figure 1). Apparent specific binding was the difference between total binding and apparent NSB. Apparent NSB was defined as NSB measured independently without α4β2 nAChR and equaled α/(1+α)*(total [^3^H]EB). The Hill equation (Eq. (1)) for characterizing binding data by fitting with SigmaPlot 11 was:

(1) $$y=A_{0}∕\left(1+{\left(K_{0.5}∕x\right)}^{n}\right)$$

Data were generated with the following parameter values published by our laboratory [18] unless otherwise stated: *K*_d1 _= 0.013 nM and *K*_d2 _= 12 nM for [^3^H]EB; *K*_i1 _= 0.84 and *K*_i2 _= 775 nM for nicotine; fraction of R1T = 0.84; fraction of R2T = 0.16 (see Figure 1 for notation). When a R1T concentration is stated, the corresponding R2T concentration is implied.

### Statistics

The one site models for saturation binding and competition data were simpler cases of the two sites models, making these two types of models nested [7]. Qualities of fit of the two types of models, therefore, were compared with the F-test [49]. The level of significance for hypothesis testing was 0.05. The confidence level for a confidence interval (CI) was 95%. CIs for dissociation constants and average *p *values were based on logarithmic values.

## Results

### Effects of ligand depletion and NSB on saturation binding to two specific sites

**Figure 2:**
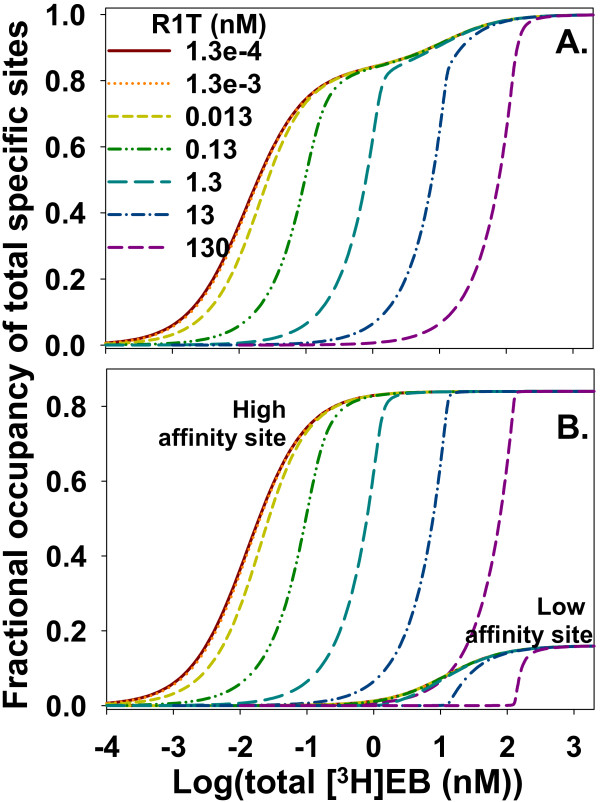
**Ligand depletion shifts the binding curve rightward and obscures distinct features of the two binding sites**. **A**. Fractional occupancy of total specific binding sites is shown at various concentrations of R1T. Increasing total binding sites increases ligand depletion, which shifts the total binding curve rightward. Ligand depletion also distorts the two sigmoidal features arising from binding to the high and low affinity sites. **B**. The fractional occupancies of high and low affinity sites are shown separately. The legend in A applies to B. Fractional occupancies of the low affinity site are clustered in the lower right corner of the plot. Only lines from R1T values of 13 and 130 nM are distinct for the low affinity site. The leftmost line from the low affinity site arises from overlap of the lines from the first five concentrations of binding sites. Binding was calculated with two sites model_free_.

The two sites model_free _generated errorless binding data using free [^3^H]EB as the independent variable to investigate how ligand depletion without NSB affected saturation binding behavior. Increasing the concentration of binding sites increased ligand depletion, shifted the total binding curve to the right, increased the steepness of the curve, and obscured the distinctive contour of the low affinity binding site (Figure 2A). The binding contour of the high affinity site began shifting noticeably to the right and showed an increasingly sharp bend at [^3^H]EB = R1T as R1T increased beyond *K*_d1 _(0.013 nM) (Figure 2B). The binding contour of the low affinity site started shifting rightward as R1T approached *K*_d2 _(12 nM). The rightward shift in the binding curves with ligand depletion means that relying on K_0.5 _as an estimate of *K*_d _overestimates dissociation constants. Eq. (2)

(2) $$K_{\text{d1}}={\text{K}}_{\text{0}\text{.5,high}}-\left(\text{R}1\text{T}∕2\right)$$

correctly estimated *K*_d1 _from the half-maximum for the high affinity site (K_0.5, high_) when K_0.5, high _was distinct [5,50]. Eq. (2), however, became increasingly difficult to use as rightward shift of the binding curve from the high affinity site led to overlap with the binding curve from the low affinity site.

**Figure 3:**
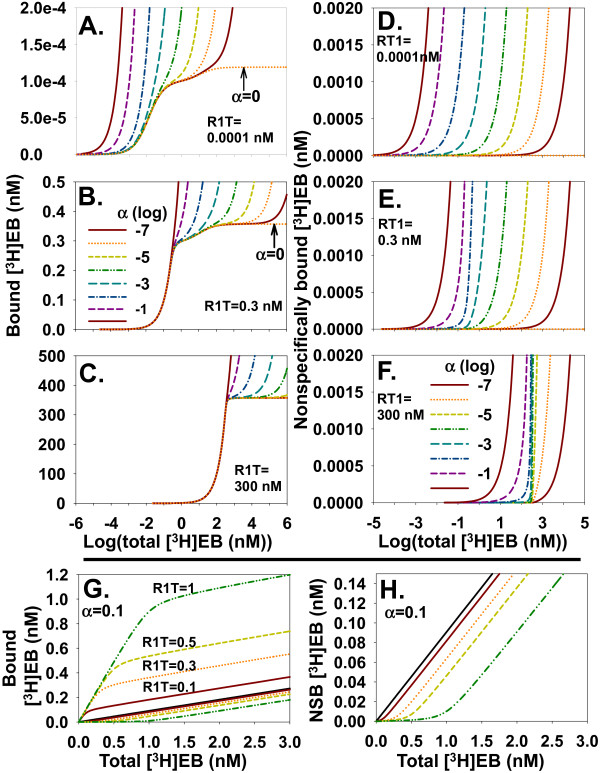
**NSB depends on the extent of ligand depletion and cannot be calculated from apparent NSB**. **A**. Total binding is shown when ligand depletion is negligible with increasing values of α ranging from 0 to 1 (integer log values of α from -7 to 0; legend for A-C in B). α = 0 is line with horizontal plateau at large concentration of [^3^H]EB. **B **and **C**. Similar to A except ligand depletion is substantial (R1T = 0.3 nM) or extreme (R1T = 300 nM). The effects of a particular α value on total binding depend on the extent of ligand depletion. **D**, **E**, **F**. NSB on expanded *y*-scales shows how increasing ligand depletion affects NSB (legend for α in F; integer log values from -7 to 0). **G**. Specific binding is not calculated correctly as the difference between total binding and apparent NSB when ligand depletion is significant. Solid black line: apparent NSB obtained without α4β2 nAChR. Lines for total binding at increasing concentrations (nM) of R1T and, therefore, increasing ligand depletion appear above apparent NSB. Corresponding lines for NSB when α = 0.1 are below apparent NSB. **H**. NSB (same code for lines as in G) and apparent NSB (solid line) from G are shown with an expanded *y*-scale. Binding in this figure was calculated with two sites model_free_.

The two sites model_free _generated errorless binding data with both ligand depletion and NSB to investigate how combining NSB with ligand depletion affected binding behavior. The effect of NSB depended on the extent of ligand depletion. With negligible ligand depletion at R1T = 0.0001 nM (Figure 3A), NSB with α = 10^-6 ^started obscuring the binding contour from the low affinity site. NSB with α = 10^-3 ^obscured binding to the high affinity site. With significant ligand depletion at R1T = 0.3 nM (Figure 3B), NSB with α = 10^-4 ^obscured the binding contour from the low affinity site. With extreme ligand depletion at R1T = 300 nM (Figure 3C), the contributions to total binding from the high affinity site and the low affinity site were not distinct even without NSB. NSB with α = 1 obscured specific binding to the high affinity site. Increasing ligand depletion also affected how NSB depended on total [^3^H]EB concentration. The leftward shift in NSB with each log unit increase in α was relatively uniform when ligand depletion was negligible at R1T = 10^-4 ^nM (Figure 3D). The leftward shift in NSB with each log unit increase in α, however, became nonuniform when ligand depletion became large (Figure 3E; R1T = 0.3 nM) or extreme (Figure 3F; R1T = 300 nM).

### Modeling specific binding and NSB as total binding

**Figure 4:**
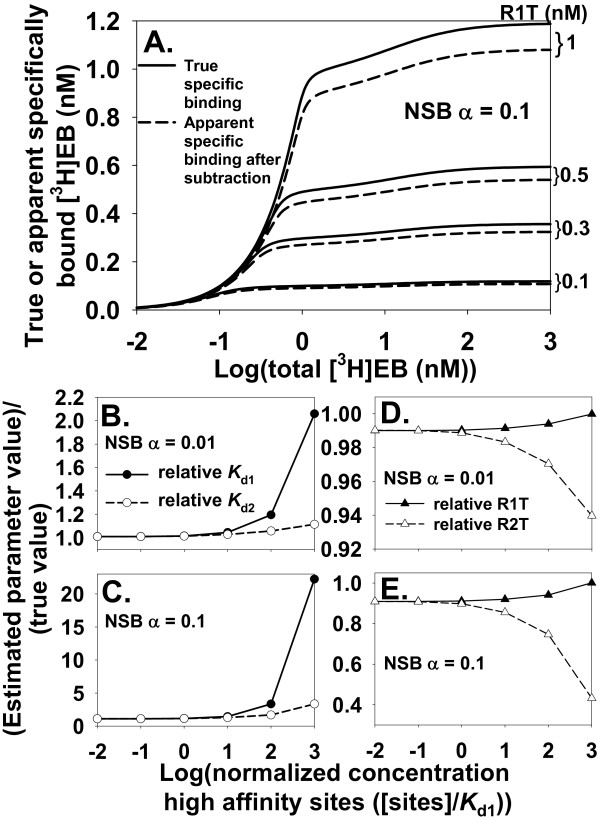
**Calculating specific binding by subtracting apparent NSB from total binding of [^3^H]EB to α4β2 nAChR leads to errors in estimating dissociation constants and binding site concentrations**. **A**. Apparent specific binding (dashed lines) calculated by subtracting apparent NSB (α = 0.1) from total binding is less than the true specific binding (solid lines). These errors in specific binding lead to errors in estimating *K*_d1 _and *K*_d2 _(**B **and **C**) and R1T and R2T (**D **and **E**) from a two sites model that includes ligand depletion but excludes NSB. B and D were obtained with α = 0.01; C and E were obtained with α = 0.1. The *x*-axis in B-E is an index of ligand depletion. Binding data shown or used in this figure were calculated with two sites model_free_.

How can dissociation constants be estimated when both ligand depletion and NSB contribute significantly to [^3^H]EB binding? An effective approach when ligand depletion is negligible is to calculate specific binding as the difference between total binding and NSB measured without α4β2 nAChR (apparent NSB). In accord with a one binding site model including ligand depletion and NSB [19], this approach was incorrect when ligand depletion was significant (Figure 3G and 3H). NSB shifted rightward from the apparent NSB as R1T increased because increasing R1T decreased the free [^3^H]EB concentration for a given total concentration of [^3^H]EB. Subtracting apparent NSB from total binding led to calculated specific binding that was shifted rightward and downward compared to true specific binding (Figure 4A). This effect led to overestimating the values of *K*_d1 _and *K*_d2 _(Figure 4B and 4C), overestimating R1T (Figure 4D and 4E), and underestimating R2T (Figure 4D and 4E) as ligand depletion increased. The difference between total binding and NSB equals specific binding by definition. These results, however, showed NSB when α4β2 nAChR was present was not equal to NSB when α4β2 nAChR was absent (apparent NSB). Specific binding, therefore, did not equal the result of subtracting apparent NSB from total binding. This inequality arose because NSB with α4β2 nAChR present did not equal apparent NSB when ligand depletion was significant. This observation has been made previously for a one site model [19].

Specific binding and NSB of [^3^H]EB and α4β2 nAChR needed to be modeled together as total binding using the two sites model_total_. This conclusion was consistent with the findings from a general one site model [19]. Accuracy of the two sites model_total _for calculating saturation binding data was tested by comparing predicted [^3^H]EB binding to [^3^H]EB binding calculated with two sites model_free_. The concentration of bound [^3^H]EB calculated by the two methods agreed to at least fourteen significant digits across this range of parameters: 10^-6 ^nM ≤ R1T ≤ 10^4 ^nM and 0 ≤ α ≤ 10^2 ^with 10^-6 ^nM ≤ [^3^H]EB_free _≤ 10^6 ^nM. These results confirmed the accuracy of the binding calculations using the two sites model_total_.

### Potential for failing to identify low-affinity specific binding when modeling only total saturation binding

An important role for the two sites model_total _is to estimate dissociation constants and binding site concentrations from noisy binding data. These estimates, however, are valid only when the two sites model_total _fits data better than does the one site model_total _according to statistical testing. Under what circumstances are binding data from the two sites of α4β2 nAChR adequately explained by the one site model_total_? In these situations, specific binding to the low affinity site is indistinguishable from high-affinity specific binding, NSB, or noise. On the other hand, what circumstances favor identifying the low-affinity specific binding site?

Deriving a computational expression for NSB from the general expression for binding to a single site suggested potential confusion between low-affinity specific binding and NSB as defined in Figure 1 (symbols similar to Figure 1):

(3) $$K_{\text{d}}=\left(\frac{\text{R}}{\text{RL}}\right)*{\text{L}}_{\text{f}}$$

(4) $$\text{R}{\text{L}}_{\text{NSB}}=\left(\frac{{\text{R}}_{\text{NSB}}}{K_{\text{d,NSB}}}\right)*{\text{L}}_{\text{f}}=\left(\frac{\text{R}{\text{T}}_{\text{NSB}}}{K_{\text{d,NSB}}}\right)*{\text{L}}_{\text{f}}$$

(5) $$\text{R}{\text{L}}_{\text{NSB}}=\text{NSB = }\alpha \text{*}{\text{L}}_{\text{f}}$$

where *α *= (RT_NSB_/*K*_d, NSB_) and R_NSB _= RT_NSB _by the definition of homogeneous NSB. If NSB arises from a collection of heterogeneous sites, then

(6) $$\text{R}{\text{L}}_{\text{NSB,total}}=\left(\frac{{\text{R}}_{\text{NSB,1}}}{K_{\text{d,NSB,1}}}+\frac{{\text{R}}_{\text{NSB,2}}}{K_{\text{d,NSB,2}}}+\cdot \cdot \cdot \right)*{\text{L}}_{\text{f}}$$

(7) $$\text{R}{\text{L}}_{\text{NSB,total}}=\left(\frac{\text{R}{\text{T}}_{\text{NSB,1}}}{K_{\text{d,NSB,1}}}+\frac{\text{R}{\text{T}}_{\text{NSB,2}}}{K_{\text{d,NSB,2}}}+\cdot \cdot \cdot \right)*{\text{L}}_{\text{f}}$$

(8) $$\text{R}{\text{L}}_{\text{NSB,total}}=\text{NSB}=\alpha *{\text{L}}_{\text{f}}$$

By analogy with these derivations, binding to the low affinity site also can be modeled as constant*L_f_, similar to NSB, when R2≃R2T. On the other hand, low-affinity specific binding behaves differently from NSB when the approximation R2≃R2T fails. This approximation most likely fails as total [^3^H]EB approaches its maximum concentration ([^3^H]EB_max_) in a saturation binding experiment. In contrast and by definition, R_NSB_≃RT_NSB _is valid for NSB; and NSB equals α*L_f _at any [^3^H]EB_max_. When [^3^H]EB_max _is sufficiently small that R2≃R2T is valid for the low-affinity specific binding site, the two sites model_total _does not fit significantly better than one sites model_total_. This outcome supports the incorrect conclusion that a second low affinity site is not present. These observations led to the hypothesis that modeling total saturation binding data with ligand depletion and NSB can blur the important biological distinction between low-affinity specific binding and NSB for [^3^H]EB and α4β2 nAChR.

### Three approaches to characterizing the low-affinity specific binding site with saturation binding

To test this hypothesis, the one site model_total _was compared to the two sites model_total _by fitting noisy total binding data from the two sites model_free _with zero NSB (α = 0). The data (60 data points and 20 total concentrations of [^3^H]EB) with R1T = 0.13 nM and [^3^H]EB_max _= 2 nM were generated with the two sites model_free _and an unrealistically large maximum signal-to-noise ratio (S/N) of 13,300 (SD = 1 × 10^-5 ^nM). The two sites model_total _fitted the data significantly better than the one site model_total _(*p *values of 1.5 × 10^-24^, 2.2 × 10^-22^, and 1.3 × 10^-20 ^for three trials). This result showed that fitting high precision total binding data with the two models identified low-affinity specific binding.

Reducing the precision of the data was expected to make detection of binding to the low affinity site more difficult. To test this expectation, binding data with the same R1T and [^3^H]EB_max _were generated with a tenfold smaller but still unrealistically large maximum S/N of 1,330 (SD = 1 × 10^-4 ^nM). Under these conditions, the two sites model_total _did not fit the data significantly better than the one site model_total _with five of five data sets (*p *= 0.33, 0.13, 0.24, 0.73, and 1.0). Fitting noisier data led to the misleading conclusion that only one specific binding site plus NSB satisfactorily accounted for the total binding data.

How can low-affinity specific binding be distinguished more reliably from NSB as S/N values decrease to realistic levels? Eqs. (3)-(5) suggested increasing [^3^H]EB_max _so the approximation R2≃R2T no longer would be valid near [^3^H]EB_max_. The approximation would break down because increased binding of [^3^H]EB to R2 at large values of [^3^H]EB would cause a significant decrease in R2 as [^3^H]EB approaches the increased value of [^3^H]EB_max_. To determine whether increasing [^3^H]EB_max _helped distinguish the low affinity binding site from NSB in the presence of ligand depletion, the one site model_total _and the two sites model_total _were fitted to noisy data with zero NSB and with [^3^H]EB_max _increased from 2 nM (60 data points) to 5 nM (63 data points). The maximum S/N of the data again was 1,330 (SD = 1 × 10^-4 ^nM). With [^3^H]EB_max _= 5 nM, the two sites model_total _fit better than the one site model_total _in five of five data sets (*p *= 4.6 × 10^-11^, 1.8 × 10^-9^, 2.8 × 10^-9^, 2.5 × 10^-9^, and 1.7 × 10^-12^). Increasing the data points from 60 to 63 did not account for this improved detection of low-affinity specific binding. Instead, this result was consistent with a breakdown of the approximation R2≃R2T as [^3^H]EB_max _increased, leading to better discernment of binding at the low affinity site at [^3^H]EB_max _= 5 nM compared to 2 nM.

**Figure 5:**
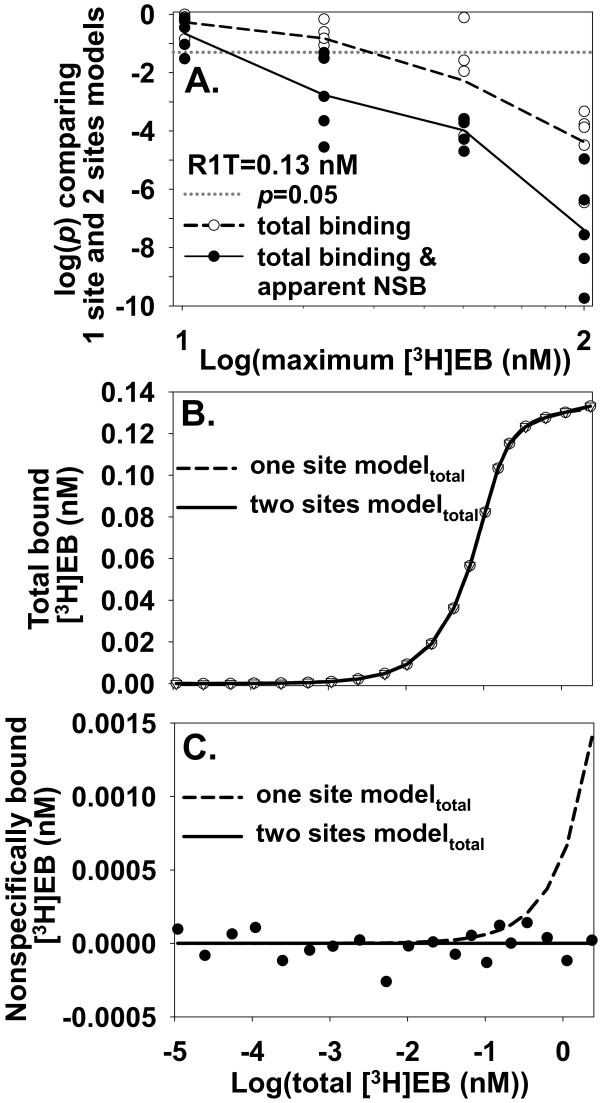
**Increasing [^3^H]EB_max _or simultaneously fitting apparent NSB helps identify low-affinity specific binding**. **A**. Data sets with maximum S/N = 36 (SD = 0.0041 nM), R1T = 0.13 nM, α = 0, and various values of [^3^H]EB_max _were fitted with one site model_total _and two sites model_total_. The *y*-axis shows *p *values from comparisons. Total binding data only (○) or total binding along with apparent NSB (●) were fit. Dashed and solid lines connect averages of log(*p*) values. At [^3^H]EB_max _= 100 nM and total binding only, the CIs included true values (*K*_d1_, 11.9-14.0 pM, mean = 12.9 pM, *K*_d2_, 3.4-12.2 nM, mean = 6.5 nM, R1T, 0.128-0.131 nM, mean = 0.129 nM; R2T, 0.014-0.021 nM, mean = 0.018 nM) (*n *= 5 for each CI). With [^3^H]EB_max _= 22 nM and explicitly fitting apparent NSB, CIs included true values (*K*_d1_, 10.6-13.2 pM, mean = 11.8 pM; *K*_d2_, 3.9-17.4 nM, mean = 8.2 nM; R1T, 0.126-0.131 nM, mean = 0.128 nM; R2T, 0.020-0.023 nM, mean = 0.021 nM) (*n *= 5 for each CI). **B, C**. With zero NSB and highly precise data (SD = 1 × 10^-4 ^nM), simultaneously fitting total binding data and apparent NSB helps identify low-affinity specific binding. Total binding data in B (40 points) were generated with R1T = 0.13 nM and [^3^H]EB_max _= 2.37 nM. The one site model_total _and two sites model_total _appear to fit total binding data equally well up to [^3^H]EB_max_. The two sites model_total_, however, fits apparent NSB values (●) in C significantly better than does the one site model_total_, leading to *p *= 2 × 10^-33 ^comparing models with simultaneous fitting. One site model_total_: *K*_d _= 0.014 nM, RT = 0.13 nM, α = 5.9 × 10^-4^; two sites model_total_: *K*_d1 _= 0.013 nM, *K*_d2 _= 10.5 nM, R1T = 0.013 nM, R2T = 0.022 nM, α = 1.0 × 10^-7^.

To explore whether larger values of [^3^H]EB_max _could distinguish low-affinity specific binding from NSB in data with more realistic precision, the one site model_total _and two sites model_total _were fitted to noisy data (maximum S/N = 36; SD = 0.0041 nM) and zero NSB (α = 0) (Figure 5A). When [^3^H]EB_max _was 10 nM, the two sites model_total _usually did not fit the data better than the one site model_total_. As [^3^H]EB_max _increased, the likelihood of better fitting by the two sites model_total _and the likelihood of support for the presence of the low affinity site also increased. At [^3^H]EB_max _= 100 nM with fitting _total _binding data only, the two sites model_total _fitted the data better than the one site model_total _for all trials. The increase in data points with increasing [^3^H]EB_max _did not account for this improved detection of low-affinity specific binding.

As a second potential approach, fitting apparent NSB while simultaneously fitting total binding data might help distinguish low-affinity specific binding from NSB by directly evaluating NSB. To test this hypothesis, total binding data (40 data points) and apparent NSB binding data (20 data points) were generated with the same conditions (maximum S/N = 1,300; SD = 1 × 10^-4 ^nM) that failed to distinguish the low affinity binding site with total binding data only. Simultaneously fitting total binding data (Figure 5B) and apparent NSB (Figure 5C) led to the two sites model_total _fitting the data significantly better than the one site model_total _in five of five data sets. The *p *values were vanishingly small (*p *= 6.5 × 10^-31^, 7.3 × 10^-34^, 3.2 × 10^-33^, 1.3 × 10^-28^, and 2.1 × 10^-33^). Figure 5C shows how fitting apparent NSB led to better detection of low-affinity specific binding. The one site model_total _could not fit total binding and simultaneously accurately fit the apparent NSB. In contrast, the two sites model_total _accurately fit the contribution from the low affinity site to total binding and simultaneously accurately fit the apparent NSB. With more realistic precision (maximum S/N = 36; SD = 0.0041 nM), the two sites model_total _usually fit the data better than did the one site model_total _for [^3^H]EB_max _≥ 22 nM (Figure 5A). In addition, simultaneously fitting both total binding and apparent NSB data more reliably identified low-affinity specific binding than did fitting only total binding. These results suggested that simultaneously fitting both total binding and apparent NSB could be superior to fitting only total binding for detecting low-affinity specific binding when NSB was negligible.

**Figure 6:**
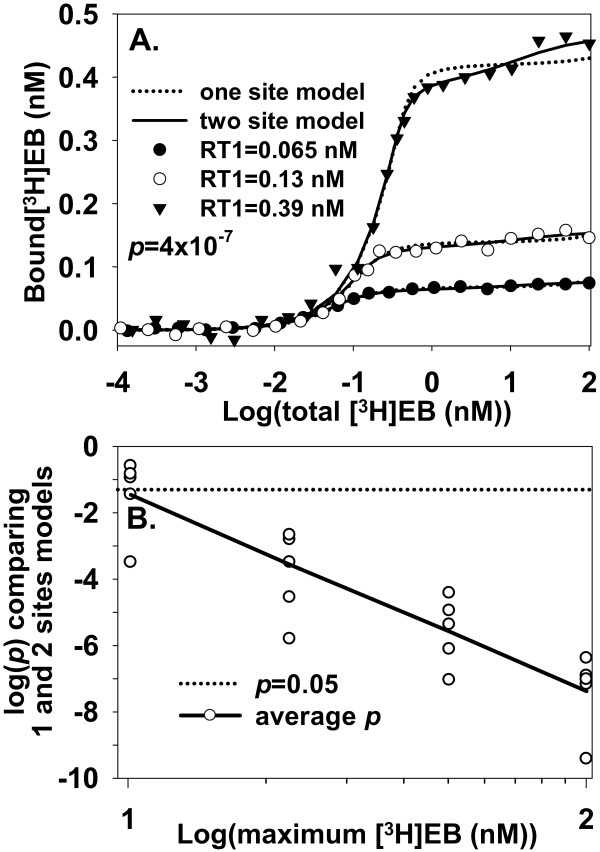
**Simultaneously fitting binding data from several different concentrations of α4β2 nAChR identifies low-affinity specific binding**. **A**. The one site model_total _and two sites model_total _with [^3^H]EB_max _= 100 nM were fitted to noisy binding data with R1T = 0.065, 0.13, and 0.39 nM and α = 0. SD for noise at the R1T values was 0.002, 0.004, and 0.012, respectively (maximum S/N ratio≃36). The two sites model_total _shows a significantly better fit (*p *= 4 × 10^-7^). **B**. The *y*-axis shows *p *values for comparisons of one site model_total _and two sites model_total _from various [^3^H]EB_max _values. The fitted data sets are analogous to the data sets shown in A; *p*-values from 5 data sets appear at each *x*-coordinate. Solid lines connect averages of log(*p*) values. The CIs of estimates of *K*_d1 _(9.2-18.5 pM; mean = 13.0 pM) and *K*_d2 _(1.0-1400 nM; mean = 38 nM) and R2T (0.015-0.66; mean = 0.34) (*n *= 5 for each CI) include true values.

A third approach for potentially distinguishing low-affinity specific binding from NSB is based on how NSB varies with α4β2 nAChR concentration. Suppose, in an idealized case, that NSB arises solely from sources (e. g., walls of a test tube, surface of a glass filter, or a constant volume of cell membranes) that are independent of α4β2 nAChR. The independence of NSB from α4β2 nAChR suggests the hypothesis that varying α4β2 nAChR concentration helps distinguish low-affinity specific binding from NSB when ligand depletion is significant. Variation in α4β2 nAChR concentration could arise by injecting different amounts of cRNA into oocytes or by transfecting different amounts of cDNA into cells. To test this hypothesis, the one site model_total _and two sites model_total _with implicit fitting of NSB were fitted to noisy [^3^H]EB binding data (maximum S/N = 36) generated at three different concentrations of α4β2 nAChR and with zero NSB (Figure 6A). The two sites model_total _consistently fit the data better than the one site model_total _for [^3^H]EB_max _≥ 22 nM (Figure 6B). In contrast, [^3^H]EB_max _in the range of 100 nM was needed when the same numbers of data points were generated under similar conditions from a single α4β2 nAChR concentration (Figure 5A). These results suggested that simultaneous fitting of data from various α4β2 nAChR concentrations, when NSB is independent of α4β2 nAChR concentration, could help distinguish binding to the low affinity binding site better than fitting data from a single α4β2 nAChR concentration.

Potentially, both sources independent of α4β2 nAChR concentration and sources correlated with α4β2 nAChR concentration might contribute significantly to NSB. The equation describing NSB in this case needs to include a component independent of (RL_NSB, indep_) and a component dependent on α4β2 nAChR concentration (RL_NSB, dep_). Based on Eqs. (3)-(5) and if RT_NSB, dep _is directly proportional to α4β2 nAChR, the relationship between NSB and free ligand becomes:

(9) $$\text{R}{\text{L}}_{\text{NSB,indep}}+\text{R}{\text{L}}_{\text{NSB,dep}}=\left(\frac{{\text{R}}_{\text{NSB,indep}}}{K_{\text{d,NSB,indep}}}+\frac{{\text{R}}_{\text{NSB,dep}}}{K_{\text{d,NSB,dep}}}\right)*{\text{L}}_{\text{f}}$$

(10) $$\text{R}{\text{L}}_{\text{NSB,indep}}+\text{R}{\text{L}}_{\text{NSB,dep}}≅\left(\frac{\text{R}{\text{T}}_{\text{NSB,indep}}}{K_{\text{d,NSB,indep}}}+\frac{\text{R}{\text{T}}_{\text{NSB,dep}}}{K_{\text{d,NSB,dep}}}\right)*{\text{L}}_{\text{f}}$$

(11) $$\text{R}{\text{L}}_{\text{NSB,indep}}+\text{R}{\text{L}}_{\text{NSB,dep}}≅\left(\frac{\text{R}{\text{T}}_{\text{NSB,indep}}}{K_{\text{d,NSB,indep}}}+\frac{\beta *\left[\alpha 4\beta 2\right]}{K_{\text{d,NSB,dep}}}\right)*{\text{L}}_{\text{f}}$$

(12) $$\text{NS}{\text{B}}_{\text{total}}=\text{R}{\text{L}}_{\text{NSB,indep}}+\text{R}{\text{L}}_{\text{NSB,dep}}≅\left(\alpha_{\text{ind}}+\alpha_{\text{dep}}*\left[\alpha 4\beta 2\right]\right)*{\text{L}}_{\text{f}}$$

Eq. (12) for NSB_total _or other expressions for RT_NSB, dep _can be incorporated into binding equations (Figure 1) when the low affinity binding site is investigated with various α4β2 nAChR concentrations and binding models.

### Characterizing the low-affinity specific binding site by ligand depletion

How does combining NSB with ligand depletion affect the interpretation of saturation binding with ligand depletion? Without ligand depletion, large NSB tended to overwhelm the signal from the low affinity site when total and free [^3^H]EB were high enough to populate the low affinity binding site (Figure 3A). Conditions leading to ligand depletion, however, would increase the concentration of the low affinity site, reduce free [^3^H]EB and NSB, and lead to relatively more binding to the low affinity site than to NSB. With α = 0.1 and R1T = 0.00013 nM (negligible depletion), the ratio R2L/NSB was 1.1 × 10^-5 ^at [^3^H]EB = 12 nM and 4.4 × 10^-6 ^at [^3^H]EB = 50 nM. As expected, NSB overwhelmed the signal from the low affinity site at and above [^3^H]EB = *K*_d2_, which was the minimal concentration range needed to significantly populate the low affinity site. In contrast, with R1T = 20 nM (substantial depletion) and the low affinity site starting to participate in ligand depletion, the ratio R2L/NSB was much larger: 3.2 at [^3^H]EB = 12 nM and 1.0 at [^3^H]EB = 50 nM.

To test this promising usefulness for ligand depletion, noisy data (maximum S/N = 50 at each R1T) with α = 0.1 and significant ligand depletion at three values of R1T (0.13, 3, and 20 nM; [^3^H]EB_max _= 0.15, 3.6, and 24 nM) were fitted by the one site model_total _and the two sites model_total_. The two sites model_total _fit the data better in ten of ten trials and produced CIs that included the true values for the parameters (*K*_d1 _= 0.0133 nM, CI = 0.0120-0.0149 nM; *K*_d2 _= 11.9 nM, CI = 9.0-15.8 nM; fraction of low affinity site = 0.180, CI = 0.156-0.204; α = 0.098, CI = 0.092-0.103). To test the effect of simultaneously fitting apparent NSB, noisy data (maximum S/N = 50) with α = 0.1 at three values of R1T (0 nM for apparent NSB alone, 0.13, and 20 nM) were fitted by the one site model_total _and the two sites model_total_. The two sites model_total _fit the data better in ten of ten trials and produced CIs including the true values for the parameters (*K*_d1 _= 0.0123 nM, CI = 0.0097-0.0156 nM; *K*_d2 _= 31.8 nM, CI = 6.5-155 nM; fraction of low affinity site = 0.291, CI = 0.133-0.450; α = 0.0997, CI = 0.0987-0.101). These results suggested that increasing ligand depletion might be useful for detecting and characterizing the low affinity site when NSB is significant in saturation binding data.

### Effects of ligand depletion and NSB on homologous competition

To investigate effects of ligand depletion and NSB on homologous competition, a two sites model_free _and a two sites model_total _were developed using concentration of free or total cold EB as the independent variable (Figure 1B). Calculations of total binding using the two sites model_total _agreed with calculations with two sites model_free _to at least fourteen significant digits. The ranges of parameters tested were 1 × 10^-6 ^nM ≤ R1T ≤ 1 × 10^4 ^nM and 0 ≤ α ≤ 20 with 1 × 10^-6 ^nM ≤ [^3^H]EB_total _≤ 1 × 10^6 ^nM. These results confirmed the accuracy of modeling homologous competition using total cold EB concentration as the independent variable.

**Figure 7:**
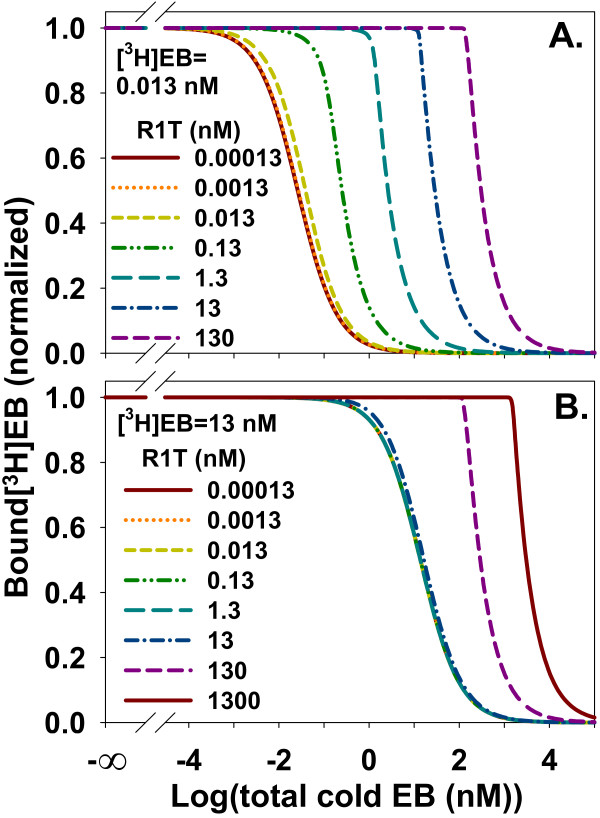
**Ligand depletion affecting homologous competition data shifts IC_50 _rightward and distorts the sigmoidal shape of the competition curve**. **A**. Homologous competition of 0.013 nM [^3^H]EB and α4β2 nAChR at various concentrations of R1T. **B**. Homologous competition of 13 nM [^3^H]EB and α4β2 nAChR. Compared with A, the larger [^3^H]EB concentration leads to an initially larger IC_50 _at small R1T (Eq. (13)) and a larger R1T concentration needed for the onset of significant ligand depletion. The curves for the five smallest R1T values overlap and form the leftmost solid curve. The competition curves of both A and B are distorted away from sigmoidal shape as ligand depletion increases.

Increasing ligand depletion by increasing R1T changed the appearance of homologous competition data using 0.013 nM [^3^H]EB, which equaled the *K*_d _for the high affinity binding site (Figure 7A). At R1T = 0.00013 nM, ligand depletion was negligible. The binding curve was symmetric about IC_50 _= 0.02612 nM with a Hill coefficient of -0.9995. The *K*_d _calculated from a modified Cheng-Prusoff equation for homologous competition [51], which ignores ligand depletion:

(13) $${\text{IC}}_{\text{50}}={[}^{3}\text{H}]\text{EB + }K_{\text{d}}$$

was 0.01316 nM, close to the value of *K*_d _for the high affinity site. Increasing ligand depletion distorted the competition curve away from a sigmoidal shape and shifted the curve rightward. The curve at R1T = 130 nM was asymmetric about IC_50 _= 306 nM and did not follow Eq. (13). When [^3^H]EB was increased to 13 nM, [^3^H]EB concentration controlled IC_50 _when ligand depletion was negligible, agreeing with Eq. (13) (Figure 7B). IC_50_, therefore, remained about 13 nM for R1T < 13 nM. Increasing ligand depletion shifted IC_50 _rightward when R1T ≥ 13 nM and made the homologous competition curves asymmetric around IC_50_. These results showed that increasing ligand depletion in homologous competition data shifted IC_50 _rightward and caused asymmetric curves around IC_50_.

**Figure 8:**
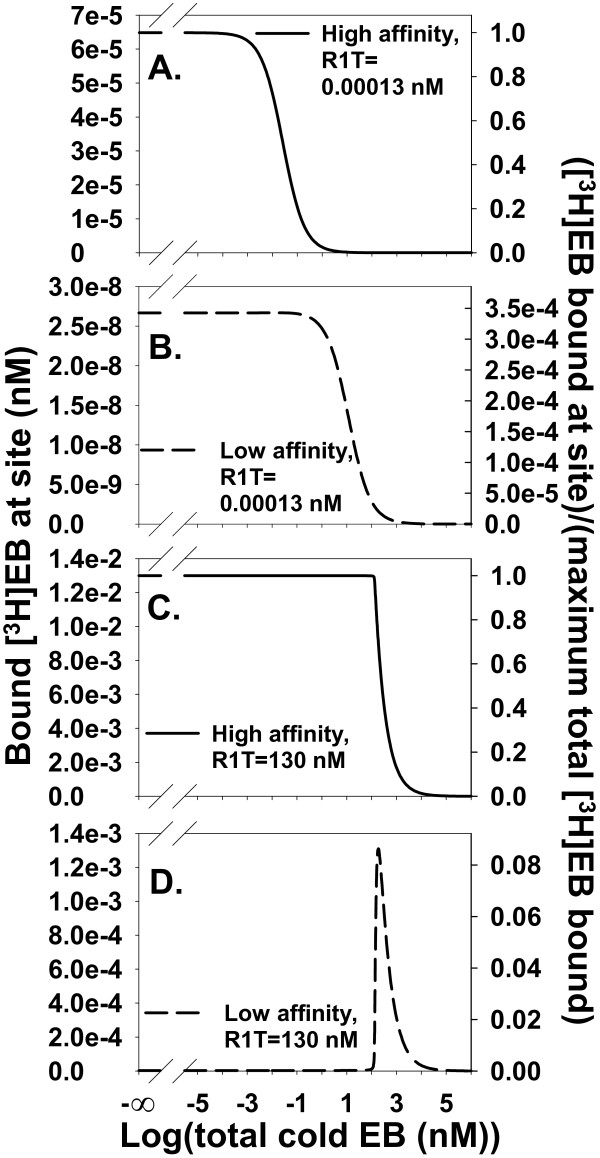
**Homologous competition of [^3^H]EB at the low affinity site is substantially different from competition at the high affinity site when ligand depletion is significant**. **A **and **B**. Homologous competition from the high affinity site (A) and the low affinity site (B) with [^3^H]EB = 0.013 nM and R1T = 0.00013 nM leads to negligible depletion of [^3^H]EB. The competition curves are sigmoidal. **C **and **D**. [^3^H]EB = 0.013 nM and R1T = 130 nM lead to significant ligand depletion. Competition at the high affinity site (C) with ligand depletion is a distorted sigmoid curve similar to the total competition curves at high ligand depletion in Figure 7. In contrast, competition at the low affinity site (D) is a peak with maximum binding at 190 nM cold EB. From the right-hand scales of A and B with the right-hand scales of C and D, ligand depletion changes the fractional contribution of the low affinity site to the total binding. The low affinity site contributes less than 3.5 × 10^-4 ^of the maximum total binding when ligand depletion is negligible (A and B). In contrast, the low affinity site contributes more than 0.08 of the maximum total binding with significant ligand depletion (C and D).

As ligand depletion increased, its effect on binding to the high affinity site became qualitatively different from its effect on binding to the low affinity site. With negligible ligand depletion at R1T = 0.00013 nM and [^3^H]EB = 0.013 nM, homologous competition of [^3^H]EB binding to the high and low affinity sites produced similarly shaped sigmoidal competition curves (Figure 8A and 8B). With substantial ligand depletion at R1T = 130 nM and [^3^H]EB = 0.013 nM, [^3^H]EB binding to the high affinity site acquired a sharp shoulder but continued to decrease monotonically with increasing cold EB (Figure 8C). At the low affinity site, substantial ligand depletion produced an asymmetric peak of [^3^H]EB binding (Figure 8D).

### Characterizing the low-affinity specific binding site with homologous competition when NSB is negligible

**Figure 9:**
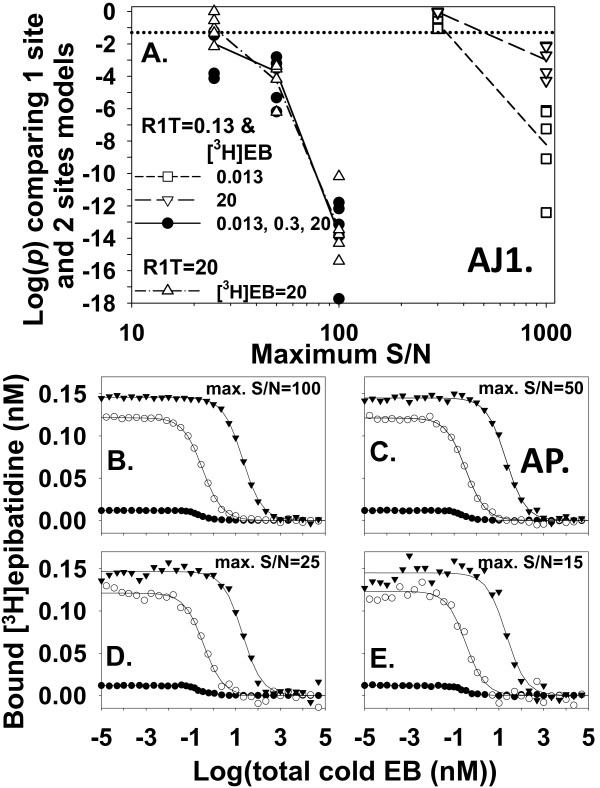
**Data exploring a wide range of fractional occupancies of both binding sites help identify the low affinity binding site with homologous competition data with zero NSB**. **A**. The *p*-values comparing one site model_total _and two sites model_total _depend on the maximum S/N in the homologous competition data. With R1T = 0.13 nM, single concentrations of [^3^H]EB with ([^3^H]EB = 0.013 nM; □; average log(*p*), short dashed line) or without ([^3^H]EB = 20 nM;∇; average log(*p*), long dashed line) ligand depletion require highly precise data (maximum S/N > 300) to consistently achieve *p *< 0.05 (*p *= 0.05, dotted line). When [^3^H]EB and R1T are 20 nM (Δ; average log(*p*), dash-dot line) and ligand depletion is significant, less precise data are needed to consistently achieve *p *< 0.05. With the same number of data points (114 points), simultaneous fitting of data from concentrations of [^3^H]EB at 0.013, 0.3, and 20 nM with R1T = 0.13 nM (●; average log(*p*), solid line) also needs less precise data to consistently achieve *p *< 0.05. Number of trials at each concentration and S/N value was 5. Estimates of dissociation constants and binding site concentrations are not significantly different from true values when S/N = 50. The CIs of *K*_d1 _(9.6 - 13.8 pM; mean = 11.5 pM) and *K*_d2 _(1.9-22.0 nM; mean = 6.5 nM) and CIs of R1T (0.126-0.131; mean = 0.128) and R2T (0.0198-0.0315; mean = 0.0256) (*n *= 5 for each CI) included the true values. **B**, **C**, **D**, and **E**. The two sites model_free _generated noisy homologous competition data sets with R1T = 0.13 nM; [^3^H]EB = 0.013 (●), 0.3 (○), and 20 nM (▼); and maximum S/N = 100 (B), 50 (C), 25 (D), and 15 (E). Fitting the one site model_total _and two sites model_total _to these types of data sets produced *p *values in A. Lines shown are fits of two sites model_total_.

How well can homologous competition data with ligand depletion identify the low affinity binding site? Comparing fits from the one site model_total _and two sites model_total _to noisy data from a single [^3^H]EB concentration reliably achieved this goal only with highly precise data (maximum S/N = 1000) (Figure 9A). With 20 nM [^3^H]EB, [^3^H]EB occupied a large fraction (62%) of the low affinity binding site when cold EB was absent. The result with 20 nM [^3^H]EB and 0.13 nM R1T suggested that occupying both high and low affinity sites using one high [^3^H]EB concentration was insufficient to identify the low affinity site when S/N values were realistic. Figure 8C and 8D, however, suggested that combining concentrations of [^3^H]EB and binding sites on the order of *K*_d2 _might lead to a distinctive concentration dependence of [^3^H]EB binding that would identify the low affinity binding site with less precise data. Indeed, concentrations of 20 nM [^3^H]EB and 20 nM R1T reliably achieved this goal with less precise data (maximum S/N = 50) (Figure 9A). These results suggested that homologous competition data from a single [^3^H]EB concentration could identify the low affinity binding site with realistically precise data using large concentrations of [^3^H]EB and α4β2 nAChR binding sites. This approach, however, consumed large amounts of [^3^H]EB and α4β2 nAChR.

Multiple concentrations of [^3^H]EB that explored a wide range of fractional occupancies of the two binding sites might identify the low affinity binding site while consuming less [^3^H]EB and α4β2 nAChR. Improving the interpretation of homologous competition data from two binding sites by using several concentrations of radioligand has been described for a general case [7]. To test this method with [^3^H]EB and α4β2 nAChR, homologous competition data sets from [^3^H]EB concentrations of 0.013, 0.3, and 20 nM and R1T = 0.13 nM were generated (Figure 9B-E). Multiple concentrations of [^3^H]EB required less precise data and consumed less [^3^H]EB and α4β2 nAChR to identify the low affinity site than did a single large [^3^H]EB concentration (Figure 9A).

### Characterizing the low-affinity specific binding site with homologous competition when NSB is significant

**Figure 10:**
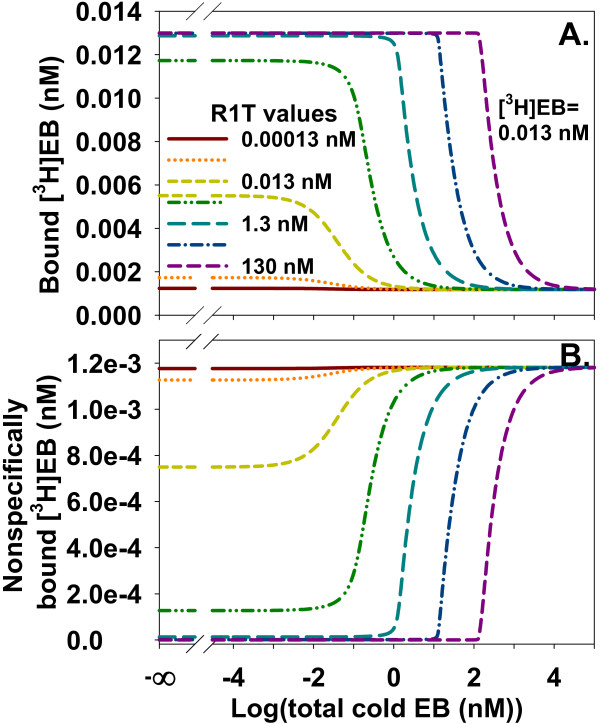
**The contribution of NSB to total binding of homologous competition data depends on the concentration of cold EB when ligand depletion is significant**. **A**. The presence of NSB (α = 0.1, [^3^H]EB = 0.013 nM) displaces the baseline of bound [^3^H]EB above the *x*-axis. Ligand depletion does not affect the size of this signal at large concentrations of cold EB, which in this case is equivalent to the apparent NSB. As anticipated from Figure 7, increasing ligand depletion in the presence of NSB shifts IC_50 _rightward and distorts the sigmoidal shape of the competition curve. R1T values increase in one log unit increments. **B**. NSB is not constant along the *x*-axis. Increasing ligand depletion with increasing R1T reveals the sigmoidal or distorted sigmoidal appearance of NSB as a function of cold EB and shifts the NSB curve rightward. Conditions and legend are the same as in A.

In practice, NSB is not zero and needs to be included in a model of homologous competition data. NSB, as expected, moved the baseline above zero at large concentrations of cold EB. Increasing ligand depletion shifted IC_50 _rightward and distorted the monotonically decreasing sigmoidal shape of the competition curve (Figure 10A). As expected from modeling of one specific binding site [19], the contribution of NSB to total [^3^H]EB binding across the range of cold EB concentration depended on the extent of ligand depletion (Figure 10B). The dependence of NSB on ligand depletion showed that simply subtracting the baseline of bound [^3^H]EB at large cold EB concentration from total bound [^3^H]EB did not accurately calculate specifically bound [^3^H]EB. Instead, and similar to saturation binding with ligand depletion and NSB, interpreting properties of specific binding of homologous competition data with NSB needed fitting of total binding.

Homologous competition without NSB suggested simultaneously fitting data from several [^3^H]EB concentrations at a constant concentration of α4β2 nAChR better identified the low affinity site than did fitting data from a single [^3^H]EB concentration (Figure 9A). Applying this approach at 0.013, 0.3, and 20 nM [^3^H]EB to homologous competition with R1T = 0.13 nM and α = 0.1, however, revealed that NSB overwhelmed specific binding at 20 nM [^3^H]EB. 92% of total [^3^H]EB binding was NSB, 7% was bound to the high affinity site, and only 1% was bound to the low affinity site in the absence of cold EB.

**Figure 11:**
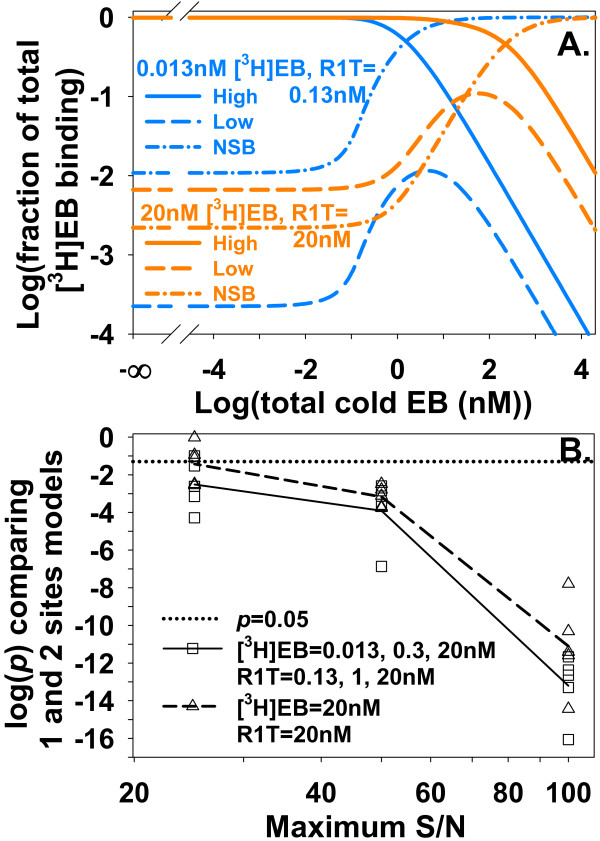
**Ligand depletion helps identify the low affinity site when NSB is significant in competition data**. **A**. Increasing concentrations of binding sites and [^3^H]EB samples a wide range of fractional contributions of the two binding sites and NSB to total binding. The combination [^3^H]EB = 0.013 nM and R1T = 0.13 nM mostly samples behavior of the high affinity site. The low affinity site contributes at most one-hundredth of the total binding; its contribution is always smaller than NSB. In contrast, the combination [^3^H]EB = 20 nM and R1T = 20 nM more effectively samples behavior of the low affinity site. The low affinity site contributes a maximum of one-tenth of the total binding and contributes more than NSB does to total binding up to about 20 nM cold EB. The *y*-axis values are calculated as Q/(R1L+R2L+NSB) where *Q *= R1L, R2L, or NSB. These results suggest this approach might adequately sample the contribution from the low affinity site to total binding during fitting of noisy data. **B**. The *p *values compare fits from one site model_total _and two sites model_total _to competition data generated with α = 0.1. One set (Δ) used [^3^H]EB = R1T = 20 nM; the second set (□), [^3^H]EB = 0.013, 0.3, and 20 nM and R1T = 0.13, 1, and 20 nM. Lines show average log(*p*). At S/N = 50 for the first set, CIs included the true values (*K*_d1 _= 0.016 nM (CI: 0.010-0.025 nM); *K*_d2 _= 14.9 nM (CI: 6.3-35 nM); R1T = 20.2 nM (CI: 19.7-20.6 nM); R2T = 4.7 nM (CI: 2.8-6.6 nM); α = 0.096 (CI: 0.091-0.100) (*n *= 5 for each CI). At S/N = 25 for the second set, CIs included the true values (*K*_d1 _= 0.012 nM (CI: 0.009-0.014 nM); *K*_d2 _= 17.0 nM (CI: 4.3-66 nM); fraction of R2T = 0.28 (CI: 0.11-0.45); α = 0.10 (CI: 0.093-0.108).

As suggested by Figures 8 and 9A, concentrations of both [^3^H]EB and R1T on the order of *K*_d2 _might help identify binding to the low affinity site. This method populates the low affinity site relative to the high affinity site and to NSB (Figure 11A). This method with [^3^H]EB and R1T at 20 nM identified binding to the low affinity site with five of five data sets at S/N = 50 and three of five data sets at S/N = 25 (Figure 11B). The consumption of a large concentration of [^3^H]EB and α4β2 nAChR at all data points, however, was an undesirable outcome.

To reduce [^3^H]EB and α4β2 nAChR consumption, both binding sites and [^3^H]EB were varied. This method could sample a wide range of fractional occupancies of the two binding sites, which suggested a potential advantage for interpreting binding to the specific sites (Figure 11A). The maximum fractional occupancies (R1L/R1T) of the high affinity site by [^3^H]EB were 0.089, 0.29, and 0.97 at [^3^]EB = 0.013, 0.3, and 20 nM and at R1T = 0.13, 1, and 20 nM. For the low affinity site, the maximum fractional occupancies (R2L/R2T) were 0.00081, 0.014, and 0.29. NSB made a greater fractional contribution to total binding than the low affinity site for all concentrations of cold EB when [^3^H]EB = 0.013 nM and R1T = 0.13 nM. With [^3^H]EB and R1T at 20 nM, however, [^3^H]EB binding by the low affinity site was greater than NSB up to 24 nM cold EB (Figure 11A). These results suggested this method might adequately sample the contribution by the low affinity site to total binding during fitting of noisy data when NSB was significant.

The method was tested by comparing one site model_total _and two sites model_total _fits to noisy data from three pairs of [^3^H]EB concentrations and binding site concentrations. The low affinity site was identified with five of five data sets with S/N = 50 and four of five data sets with S/N = 25 (Figure 11B). These results suggested that simultaneous fitting of homologous competition data from several concentrations of [^3^H]EB and binding sites has the potential to identify low-affinity specific binding in the presence of NSB.

### Potential misinterpretation of low-affinity specific binding as NSB in homologous competition binding

**Figure 12:**
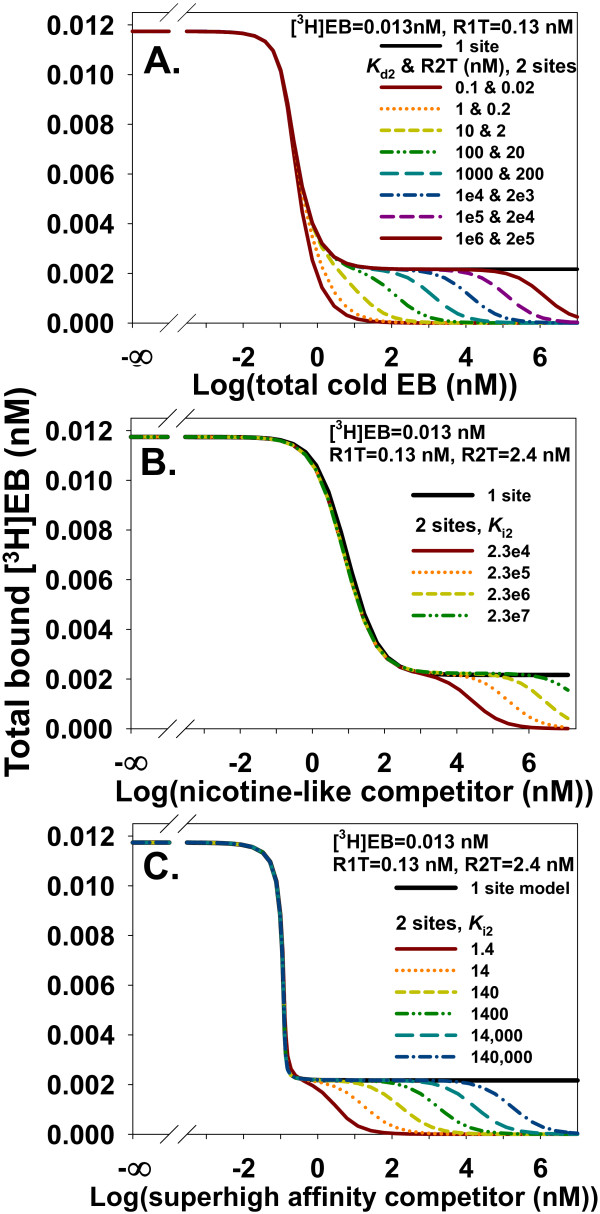
**Binding to a low affinity second binding site with hypothetically large R2T and *K*_d2 _or *K*_i2 _values mimic NSB in homologous and heterologous competition data**. **A**. The one site model_free _(α = 0.2) and two sites model_free _(α = 0) generated homologous competition data with *K*_d2 _= R2T/0.2. With increasing R2T and *K*_d2_, the two sites model_free _produces increasingly long plateaus of total bound [^3^H]EB, similar to NSB. Low-affinity specific binding is distinguished from NSB only when cold EB concentration exceeds *K*_d2_. **B**. Heterologous competition of [^3^H]EB with a nicotine-like competitor at a low affinity site can mimic NSB. The one site model_free _with α = 0.2 generated heterologous competition data with *K*_i1 _= 0.84 nM for the competitor (value for nicotine [18]). With these values and R2T = 2.4 nM and α = 0, the two sites model_total _fits these data well up to a competitor concentration of *K*_i2_. Low-affinity specific binding is distinguished from NSB only when the competitor concentration exceeds *K*_i2_. **C**. With a superhigh affinity competitor, increasing R2T and varying *K*_i2 _with heterologous competition of [^3^H]EB at a low affinity site can mimic NSB. The one site model_free _with α = 0.2 generated heterologous competition data with *K*_i1 _= 1.3 × 10^-4 ^nM. With α = 0, the two sites model_total _fits well to these data up to a competitor concentration of *K*_i2_. Low-affinity specific binding is distinguished from NSB only when competitor concentration exceeds *K*_i2_.

Even with highly precise data, Eqs. (3) to (5) suggested a possibility of misinterpreting low-affinity specific binding as NSB in homologous competition data when only fitting total binding data. A low affinity, second specific binding site with a large relative concentration could mimic NSB as long as R2≈R2T over the range of cold EB concentration. Although a large relative concentration of the second binding site was not observed from expression of α4β2 nAChR in oocytes [18], such a condition potentially could arise in a different heterologous expression system. The potential for confusing low-affinity specific binding and NSB was explored by comparing homologous competition data from a one site model_free _with α = 0.2 with data from a two sites model_free _with α = 0 and *K*_d2 _= R2T/0.2. As R2T and *K*_d2 _increased, the upper limit of cold EB concentration for which R2 R2T remained valid also increased. The data from the two sites model_free _with zero NSB, therefore, displayed increasingly long plateaus mimicking NSB at large concentrations of cold EB. The long plateaus, however, arose from specific binding to the low affinity α4β2 nAChR binding site and not from NSB. Figure 12A suggested that homologous competition data at a single [^3^H]EB concentration might not distinguish binding to a low affinity site from NSB unless either the maximum concentration of cold EB exceeded *K*_d2 _or NSB was measured without α4β2 nAChR.

### Heterologous competition with ligand depletion and NSB

Homologous competition is a specific case of the more general case of heterologous competition, for which the dissociation constants of the radioligand and the heterologous competitor differ. For heterologous competition, identification of a low affinity site and estimates for dissociation constants for [^3^H]EB to high and low affinity sites typically are determined from saturation binding. In this case, inhibition constants (*K*_i1 _and *K*_i2 _in Figure 1) for the competitor and the concentration of binding sites are the only unknowns when fitting heterologous displacement data. This study focuses on how ligand depletion and NSB affects heterologous competition with high and low affinity binding sites of [^3^H]EB. In addition, this study investigates concentrations of [^3^H]EB and α4β2 nAChR that might facilitate studying the low affinity site.

**Figure 13:**
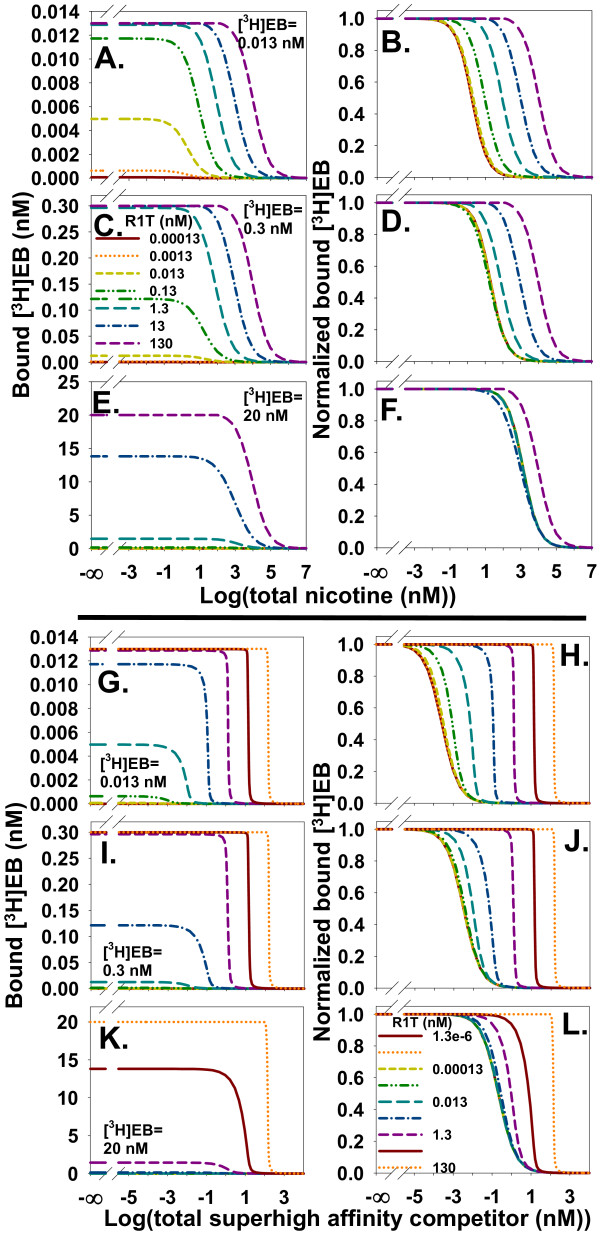
**Effects of ligand depletion on heterologous competition data depend on the relative affinity of the inhibitor**. **A-F**. Competition data for [^3^H]EB and nicotine were generated with two sites model_free _with [^3^H]EB = 0.013 (A & B), 0.3 (C & D), and 20 nM (E & F) and the R1T values shown in C. The *y*-axes of A, C, and E show total bound [^3^H]EB; *y*-axes of B, D, and F show normalized binding for comparing IC_50 _values. Data from small values of R1T are not distinguishable because of the ranges of the *y*-axis scales (A, C, and E) or because data sets overlap when rightward shifts of IC_50 _are negligible (B, D, and F). Ligand depletion shifts IC_50 _rightward; shape of the competition curve remains approximately sigmoidal. **G-L**. Competition data for [^3^H]EB and a hypothetical superhigh affinity competitor were generated with two sites model_free _with [^3^H]EB = 0.013 (G & H), 0.3 (I & J), and 20 nM (K & L) and the R1T values shown in L. The two inhibition constants *K*_i1 _and *K*_i2 _were 100-fold tighter (1.3 × 10^-4 ^and 0.12 nM) than *K*_d1 _and *K*_d2 _for [^3^H]EB. Ligand depletion shifts IC_50 _rightward and increases the maximum steepness of the negative slope of the sigmoidal shape. Hill coefficients at R1T = 130 nM are -35, -35, and -17 for [^3^H]EB = 0.013, 0.3, and 20 nM.

To determine how ligand depletion without NSB affects heterologous competition with [^3^H]EB and α4β2 nAChR, competition data at increasing concentrations of binding sites were generated with nicotine as the competitor. The dissociation constants for nicotine were 0.84 nM for the high affinity site [18] and 775 nM for the low affinity site. The inhibition constant for nicotine at the low affinity site was assigned so that *K*_i2_/*K*_i1 _for nicotine = *K*_d2_/*K*_d1 _for EB. When ligand depletion was negligible, IC_50 _values varied only slightly with binding site concentration (Figure 13A-F). The *K*_i _values derived from these IC_50 _values and the Cheng-Prusoff equation (Eq. (14)),

(14) $${\text{IC}}_{\text{50}}={[}^{3}\text{H}]\text{epibatidine}+K_{\text{i}}$$

which assumes a single binding site without ligand depletion, were close to *K*_i1 _for nicotine (0.90, 0.87, and 0.96 nM at 0.013, 0.3, and 20 nM [^3^H]EB and R1T = 0.00013 nM). As increasing ligand depletion shifted IC_50 _rightward (Figure 13A-F), the estimate of *K*_i _from the Cheng-Prusoff equation no longer closely matched *K*_i1 _for nicotine. The shape of the competition curve remained approximately sigmoidal with a Hill coefficient consistently near -1 at all levels of ligand depletion.

Although nicotine binds more weakly than [^3^H]EB to α4β2 nAChR, other ligands developed in the future, especially derivatives of EB, conceivably might bind more tightly than [^3^H]EB. To determine how ligand depletion affects heterologous competition with a superhigh affinity competitor, heterologous competition data were generated with two dissociation constants 100-fold tighter (1.3 × 10^-4 ^and 0.12 nM) than the two dissociation constants for [^3^H]EB. When ligand depletion of [^3^H]EB was negligible, IC_50 _values were independent of binding site concentration and led to slightly high estimates of *K*_i1 _(1.4 × 10^-4 ^nM) using Eq. (14); Hill coefficients were about -1 (Figure 13G-L). Increasing ligand depletion shifted IC_50 _rightward and, in contrast to nicotine, shifted Hill coefficients to strongly negative values (for example, -35 with [^3^H]EB = 0.013 nM and R1T = 130 nM). These results showed the effect of ligand depletion on the Hill coefficient depended markedly on whether the competitor bound more tightly or less tightly than [^3^H]EB.

**Figure 14:**
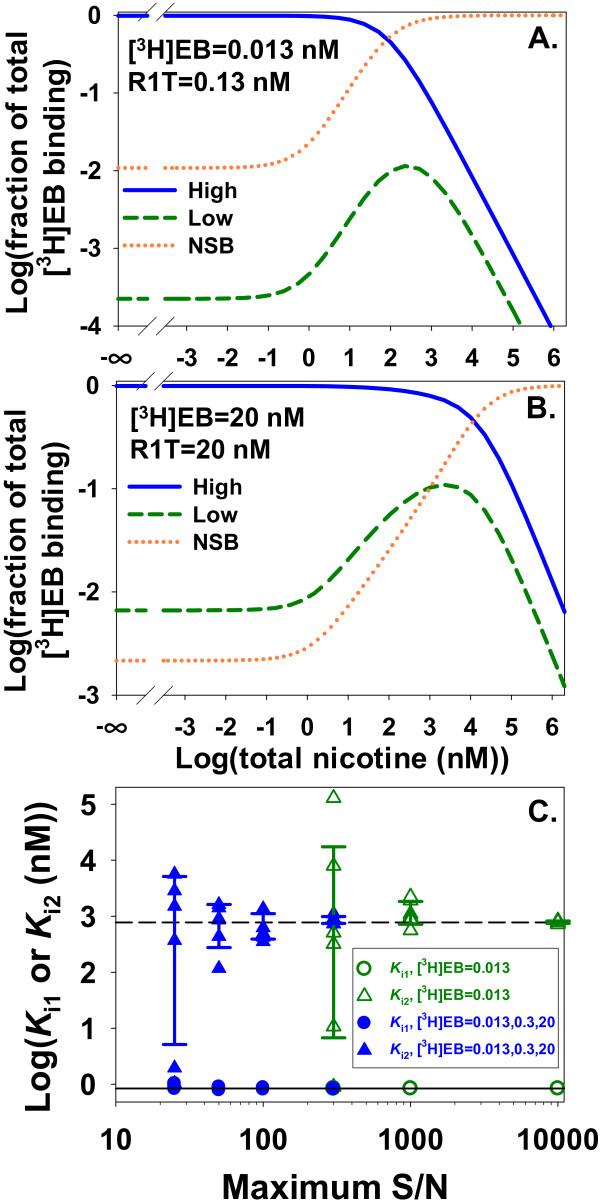
**Ligand depletion improves precision of estimated *K*_i2 _for nicotine with noisy data and NSB**. Ligand depletion improves precision of estimated *K*_i2 _for nicotine with noisy data. **A **and **B**. Increasing concentrations of binding sites and [^3^H]EB samples a wide range of fractional contributions of the two binding sites and NSB to total [^3^H]EB binding as nicotine concentration varies. In A, the combination [^3^H]EB = 0.013 nM and R1T = 0.13 nM predominantly samples interaction between [^3^H]EB and nicotine at the high affinity site. In B, the combination [^3^H]EB = 20 nM and R1T = 20 nM with substantial ligand depletion more effectively samples interaction between [^3^H]EB and nicotine at the low affinity site. The low affinity site contributes a maximum of one-tenth of total [^3^H]EB binding and contributes more than NSB does to total [^3^H]EB binding up to about 1000 nM nicotine. The *y*-axis values were calculated as Q/(R1L+R2L+NSB) where *Q *= R1L, R2L, or NSB. **C**. Noisy heterologous competition data for [^3^H]EB and nicotine with various maximum S/N were fit with two sites_total _model to estimate *K*_i1 _and *K*_i2_. The *K*_i2 _estimates shown with green Δ were derived with R1T = 0.13 nM. Modest ligand depletion and negligible occupancy by [^3^H]EB of the low affinity binding site lead to low precision of *K*_i2 _estimates at maximum S/N = 300. *K*_i2 _estimates shown with blue ▲ were derived with R1T = 0.13, 1, 20 nM. Increasing ligand depletion and occupancy of the low affinity site by [^3^H]EB lead to more precise *K*_i2 _estimates with noisier data. Error bars show standard deviations. *K*_i1 _estimates (green ○ or blue ●) are relatively independent of maximum S/N values. The number of data points (114 points) was identical in the two sets of estimates. Solid line: true *K*_i1 _(0.84 nM); dashed line: true *K*_i2 _(775 nM); α_L _= 0.1; α_B _= 0.

*K*_i2 _for a competitor potentially can be estimated with procedures analogous to procedures investigated for homologous competition. To test the approach described in Figures 9 and 11 for homologous competition, noisy heterologous competition data for nicotine and [^3^H]EB with ligand depletion and NSB were fit with the two sites_total _model (Figure 14). A single 0.013 nM concentration of [^3^H]EB with R1T = 0.13 nM did not significantly populate the low affinity site (Figure 14A). That concentration combination produced reliable estimates of *K*_i2 _only with highly precise data (maximum S/N ≥ 1000) (Figure 14C). At maximum S/N = 100, fits with competition by nicotine at the high and low affinity sites generally were not significantly better than fits with competition by nicotine at only the high affinity site (*p *> 0.05 for six of six trials). Similar to the findings in Figures 9 and 11, increasing ligand depletion and populating both the high and low affinity sites with larger concentrations of [^3^H]EB and α4β2 nAChR (Figure 14B) allowed more reliable estimates of *K*_i2 _with less precise data (Figure 14C). At maximum S/N = 15 with this approach, fits with competition by nicotine at the two [^3^H]EB binding sites generally were significantly better than fits with competition by nicotine at only the high affinity site (0.007 <*p *< 5 × 10^-10 ^for six of six trials). These results suggest that fitting data with large ligand depletion might identify the presence of nicotine competition at the low affinity site even if those data have a low S/N and an estimate of *K*_i2 _has low precision.

Similar to homologous competition data (Figure 12A), low-affinity specific binding might be misinterpreted as NSB when fitting heterologous competition data with a model of total binding. To investigate this possibility with a nicotine-like inhibitor (*K*_i1 _= 0.84 nM), heterologous depletion data from the one site model_free _with NSB (α = 0.2) were compared to data from the two sites model_total _without NSB. With R2T = 2.4 nM and various values of *K*_i2_, the two sites model_total _produced a long plateau mimicking NSB (Figure 12B). The value of *K*_i2 _at this constant value of R2T determined the length of the plateau along the *x*-axis. One log unit increase of the value of *K*_i2 _lengthened the plateau of binding to the low affinity site by one log unit. A competitor binding more tightly than [^3^H]EB to the high affinity binding site produced similar results (Figure 12C). These results suggested that binding to the low affinity site might be identified as NSB at a single [^3^H]EB concentration unless either the maximum competitor concentration was greater than *K*_i2 _or NSB was measured without α4β2 nAChR.

### Characterizing high and low affinity binding sites when NSB of a heterologous competitor is unknown

**Figure 15:**
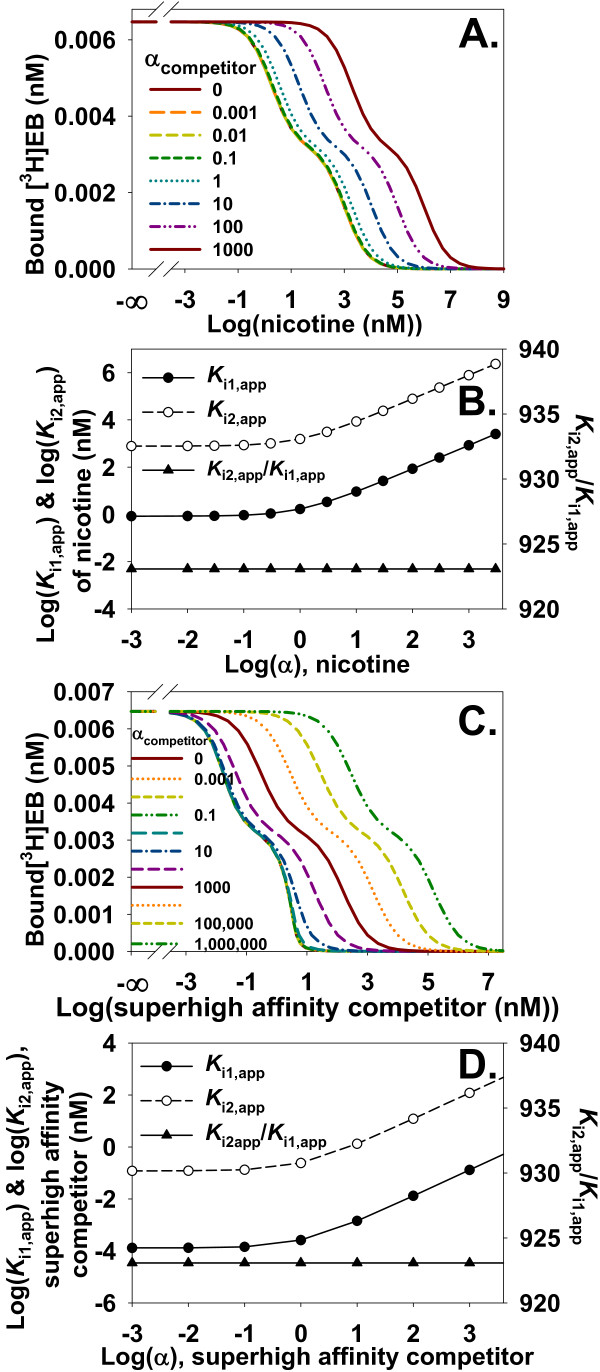
**NSB of a competitor changes *K*_i1, app _and *K*_i2, app _but not *K*_i2, app_/*K*_i1, app_**. **A**. The two sites model_free _generated competition data for [^3^H]EB and nicotine with R1T = 0.013 nM and R2T = 3.9 nM so the competition curve clearly shows the two binding sites. Increasing α_competitor _shifted the curve rightward without changing the shape of the curve. **B**. To determine how increasing α_competitor _affected apparent values of *K*_i1 _(*K*_i1, app_) and *K*_i2 _(*K*_i2, app_) with α_competitor, app _= 0, two sites model_total _was fitted to the competition curves in A. The only degrees of freedom for this fitting were *K*_i1, app _and *K*_i2, app _for nicotine. Increasing α_competitor _increases *K*_i1, app _and *K*_i2, app _but does not change *K*_i2, app_/*K*_i1, app_, a ratio reflecting the difference in free energy of nicotine binding at the two sites. **C**. Similar to A, the two sites model_free _generated competition data for [^3^H]EB and a superhigh affinity competitor with R1T = 0.013 nM, R2T = 3.9 nM, *K*_i1 _= 0.00013 nM, and *K*_i2 _= 0.12 nM. The curves clearly show the two binding sites. In contrast to A, shapes of the competition curves change as increasing α_competitor _shifts the curve rightward. **D**. Similar to nicotine, *K*_i2, app_/*K*_i1, app _for the superhigh affinity competitor does not vary with α_competitor_.

The NSB of an unlabeled competitor is not measured by heterologous competition measurements and often is assumed to be zero. The true value of α_competitor_, therefore, presents a source of uncertainty about values of inhibition constants. This uncertainty was investigated by increasing values of α_competitor _while nicotine (Figure 15A) or a superhigh affinity competitor (Figure 15C) inhibited binding of [^3^H]EB to α4β2 nAChR. As the true value of α_competitor _for nicotine increased, apparent values of *K*_i1 _(*K*_i1, app_) and *K*_i2 _(*K*_i2, app_) also increased (Figure 15B). The contours of competition curves with the superhigh affinity competitor changed as α_competitor _increased (Figure 15C), in contrast to the constant contours with nicotine. The ratio *K*_i2, app_/*K*_i1, app _for the superhigh affinity competitor, however, was invariant as α_competitor _increased (Figure 15D). The invariance of *K*_i2, app_/*K*_i1, app _at the two binding sites of α4β2 nAChR is important because the ratio represents the difference in free energy of binding at the two binding sites. This difference reflects differences in the interactions between the competitor and binding sites and structural differences between the high and low affinity binding sites. This measured free energy difference is independent of α_competitor_.

## Discussion

A model that fits total binding data as a function of total ligand can correctly interpret those data when ligand depletion and NSB are significant [19]. This approach is straightforward with one binding site. This study shows that the approach for [^3^H]EB, α4β2 nAChR, and two binding sites needs modifications for identifying binding to the low affinity site. In particular, identifying the low affinity site can be challenging because of phenomenological and computational similarities between low-affinity specific binding and NSB.

This study is novel because it shows that fitting total binding data from [^3^H]EB and α4β2 nAChR might be insufficient for characterizing the low affinity site when ligand depletion and NSB are significant. Moreover, this investigation develops four concepts for studying the low affinity binding site of α4β2 nAChR in the presence of ligand depletion and NSB that go beyond simply fitting total binding. First, binding of [^3^H]EB to the low affinity site in saturation data or homologous competition data can be misattributed to NSB. Low-affinity specific binding can be identified by using larger maximum concentrations of [^3^H]EB or cold competitor, simultaneously fitting apparent NSB, or obtaining data from multiple concentrations of α4β2 nAChR. Potential ambiguity between low-affinity specific binding and NSB arises because they share a similar appearance as long as R2≃R2T. Increasing [^3^H]EB_max _for saturation binding or increasing the maximum concentration of cold competitor for competition binding breaks this similarity by creating conditions for which R2≪R2T, R2L≃R2T, and R2B≃R2T.

Second, when NSB is significant, ligand depletion can help characterize the low affinity site. Ligand depletion in binding studies is commonly believed to be only problematic. In contrast, increasing ligand depletion by increasing α4β2 nAChR concentration beneficially reduced NSB and significantly populated the low affinity site. The result was better detection of [^3^H]EB binding to the low affinity site.

Third, directly measuring NSB without α4β2 nAChR can more reliably interpret NSB than does modeling NSB as a component of total binding in competition binding. Whether [^3^H]EB binding at a particular large concentration of competitor arises solely from NSB depends on *K*_i2 _and concentration of the low affinity site. Removing α4β2 nAChR from the assay, when feasible, is a more rigorous way than is using a large concentration of competitor to ensure that [^3^H]EB binding arises from NSB and does not involve the low affinity site of α4β2 nAChR.

Fourth, α_competitor _needs to be considered when interpreting heterologous competition data with [^3^H]EB and α4β2 nAChR because it increases *K*_i1, app _and *K*_i2, app_. The true values of *K*_i1 _and *K*_i2_, therefore, can be determined only when α_competitor _is known. Regardless of α_competitor_, however, *K*_i2, app_/*K*_i1, app _is invariant and equals *K*_i2_/*K*_i1_. This ratio can help compare structural features of the two binding sites of α4β2 nAChR. For example, variations in the ratio for a series of competitors with systematic structural variations might correlate with structural features of the two binding sites.

The findings presented in this study have limitations. First, modeling explored conditions suitable for characterizing low affinity binding that might not match conditions readily available in a laboratory. One such condition is nanomolar concentrations of α4β2 nAChR. This high range of α4β2 nAChR concentration might be more available in the future with high level heterologous expression of α4β2 nAChR. Quantitative results, such as concentration ranges that identify the low affinity site, are a reasonable but not definitive guide to conditions for studying the low affinity site of α4β2 nAChR with [^3^H]EB. For example, values of α might be substantially smaller than the values illustrating NSB in this study. With membrane homogenates from stably transfected HEK 293 cells, α was on the order of 0.001 [52]. In addition, changes in the fraction of low affinity site, as might occur with different expression conditions, will change the appearance of data. A larger fraction of low affinity site would make detection and analysis of this site easier. Second, the simulations included large numbers of data points with the goal of reliably describing binding data. Fewer data points would need higher precision in the data to identify the low affinity site and would lead to reduced precision of binding parameter estimates. Third, the properties of noise imposed on errorless data in this study do not necessarily reflect properties of real noise and uncertainties in experiments. Fourth, based on binding data from our laboratory [18], this study assumes two independent binding sites in α4β2 nAChR. Other descriptions of binding sites (for example, two cooperative binding sites, a combination of cooperative and independent binding sites, or more than two independent sites) might better describe binding data from α4β2 nAChR under other conditions. Fifth, the linear relationship between free [^3^H]EB and NSB led to the phenomenological and computational similarity between low affinity binding and NSB expressed in Eqs. (3)-(5). This linear relationship usually describes the behavior of NSB. This linear relationship might be unsuitable for some situations. For example, if NSB in the absence of specific binding is observed to be saturable [53-55], the linear relationship would need to be modified. Sixth, statistical comparisons using the F-test and *p *values between the one site model_total _and two sites model_total _were suitable because of the nested nature of the two models. In other words, the two sites model_total _contained all the features of the one site model_total _and extended those features by a second specific binding site. Other statistical methods for comparing models do not need nested models, such as Akaike's information criterion [7,56,57]. Seventh, the independent variable for the models in this study is the concentration of total ligand ([^3^H]EB for saturation binding or a cold ligand for competition). This variable usually is accurately known and was presumed to be free of uncertainty. Using the measured concentration of free ligand as the independent variable simplifies the model equations. The measured free ligand concentrations, however, will have nonnegligible uncertainty. The method of least squares might not reliably estimate parameter values when the values of the independent variable are uncertain [1,2,58].

## Conclusions

Characterizing the low affinity site potentially will contribute understanding of structure, function, and synthesis of α4β2 nAChR in native and heterologous expression systems. For example, the low affinity site might arise from an immature form of α4β2 nAChR or be involved in ligand-induced upregulation [32,59-61]. Heterologous competition data similar to Figure 12B were found with cytisine, nicotine, and acetylcholine as competitors of [^3^H]EB binding with α4β2 nAChR immunoisolated with monoclonal antibody (mAb) 295 but not with other mAbs [18]. This similarity suggests that mAb 295 might isolate a distinctive form of low affinity α4β2 nAChR. Homologous competition data might help further characterize this form of α4β2 nAChR. An intriguing possibility is that this low affinity form contributes to the biological roles of α4β2 nAChR. This study should help investigators design experiments and develop computational approaches for interpreting data from [^3^H]EB and α4β2 nAChR when ligand depletion and NSB are significant. Manipulation of maximum ligand and receptor concentrations and intentionally increasing ligand depletion are potentially helpful approaches. Extending the modeling and numerical solution method to three or more binding sites and to cooperative binding with ligand depletion and NSB is straightforward. Although applied specifically to [^3^H]EB and α4β2 nAChR, the methods should be relevant to other contexts of multiple binding sites, ligand depletion, and NSB.

## Abbreviations

CI: 95% confidence interval; EB: epibatidine; mAb: monoclonal antibody; nAChR: nicotinic acetylcholine receptor; NSB: nonspecific binding; S/N: signal to noise ratio; SD: standard deviation; [^3^H]EB: [^3^H]epibatidine.

## Competing interests

The authors declare that they have no competing interests.

## Authors' contributions

AP determined the observed binding constants describing [^3^H]epibatidine and nicotine binding to α4β2 nAChR and edited the manuscript. GW conceived the study, created the models, analyzed the simulations, and wrote the manuscript. Both authors read and approved the final manuscript.
